# Rapid intraoperative visualization of breast lesions with γ-glutamyl hydroxymethyl rhodamine green

**DOI:** 10.1038/srep12080

**Published:** 2015-07-13

**Authors:** Hiroki Ueo, Yoshiaki Shinden, Taro Tobo, Ayako Gamachi, Mitsuaki Udo, Hisateru Komatsu, Sho Nambara, Tomoko Saito, Masami Ueda, Hidenari Hirata, Shotaro Sakimura, Yuki Takano, Ryutaro Uchi, Junji Kurashige, Sayuri Akiyoshi, Tomohiro Iguchi, Hidetoshi Eguchi, Keishi Sugimachi, Yoko Kubota, Yuichiro Kai, Kenji Shibuta, Yuko Kijima, Heiji Yoshinaka, Shoji Natsugoe, Masaki Mori, Yoshihiko Maehara, Masayo Sakabe, Mako Kamiya, John W. Kakareka, Thomas J. Pohida, Peter L. Choyke, Hisataka Kobayashi, Hiroaki Ueo, Yasuteru Urano, Koshi Mimori

**Affiliations:** 1 Department of Surgery, Kyushu University Beppu Hospital, 4546 Tsurumihara, Beppu 874-0838; 2 Department of Pathology, Kyushu University Beppu Hospital, 4546 Tsurumihara, Beppu 874-0838; 3 Department of Pathology, Oita University, Yufu 879-5593, Japan; 4 Ueo Breast Surgery Hospital, Oita 870-0854, Japan; 5 Department of Digestive Surgery, Breast and Thyroid Surgery, Kagoshima University Graduate School of Medical and Dental Sciences, Kagoshima 890-8520, Japan; 6 Department of Gastroenterological Surgery, Graduate School of Medicine, Osaka University, Suita 565-0871, Japan; 7 Department of Surgery and Sciences, Graduate School of Medical Sciences, Kyushu University, Fukuoka 812-8582; 8 Graduate School of Medicine, The University of Tokyo, Tokyo 113-0033; 9 Signal Processing and Instrumentation Section, Division of Computational Bioscience, Center of Information Technology, National Institutes of Health, Bethesda, MD 20892-5624, USA; 10 Molecular Imaging Program, Center for Cancer Research, National Cancer Institute, National Institutes of Health, Bethesda, MD 20892-1088, USA; 11 Basic Research Program and CREST, Japan Science and Technology Agency, Tokyo 102-0076, Japan

## Abstract

We previously developed γ-glutamyl hydroxymethyl rhodamine green (gGlu-HMRG) as a tool to detect viable cancer cells, based on the fact that the enzyme γ-glutamyltranspeptidase (GGT) is overexpressed on membranes of various cancer cells, but is not expressed in normal tissue. Cleavage of the probe by GGT generates green fluorescence. Here, we examined the feasibility of clinical application of gGlu-HMRG during breast-conserving surgery. We found that fluorescence derived from cleavage of gGlu-HMRG allowed easy discrimination of breast tumors, even those smaller than 1 mm in size, from normal mammary gland tissues, with 92% sensitivity and 94% specificity, within only 5 min after application. We believe this rapid, low-cost method represents a breakthrough in intraoperative margin assessment during breast-conserving surgery.

Breast cancer (BC) is the most frequently diagnosed cancer in females worldwide, with about 1.38 million new cases per year^1^. Many newly diagnosed patients undergo surgery, and in Western Europe, about 60–80% of newly diagnosed BC are amenable to breast-conserving surgery (BCS)^2^. In BCS, it is extremely important to diagnose the surgical margins intraoperatively in order to prevent local recurrence and to avoid the need for additional operations. A meta-analysis suggested that the odds of local recurrence were 2.42 for positive versus negative margins in women undergoing BCS^3^. Thus, there is a need for a rapid and convenient intraoperative diagnostic method. So far, however, there is no clear guideline on intraoperative margin assessment for BCS, although various methods have been evaluated^4^,^5^,^6^,^7^,^8^. Intraoperative frozen section analysis (IFSA) is widely used because of its diagnostic accuracy. However, it is not feasible in terms of manpower, cost and time to conduct total-circumferential examination with IFSA^9^. Many surgeons and pathologists select only a few samples from the margins, for example, the nipple and distal directions and lateral margins, for IFSA. However, this inevitably results in false-negatives^9^. There are few clinical tools to identify “skeptical regions” that require detailed pathological examination.

Various techniques are available for observing the real-time dynamics of biological activity in living cells, and fluorescence-based techniques offer the advantages of rapidity, safety and convenience. Further, cell-permeable probes based on organic small molecules can be rapidly introduced into cells simply by adding them to the extracellular fluid^10^. We previously developed γ-glutamyl hydroxymethyl rhodamine green (gGlu-HMRG) as a tool to detect viable cancer cells, based on the fact that the enzyme γ-glutamyltranspeptidase (GGT) is overexpressed on membranes of various cancer cells, but is not expressed in normal tissue^3^. However, the clinical usefulness of gGlu-HMRG remained to be established. Therefore, in the present study, we focused on clinical application of gGlu-HMRG and examined its usefulness for intraoperative margin assessment during BCS.

First, we confirmed that activation of gGlu-HMRG fluorescence occurred in four types of BC cell lines (MCF7, MDA-MB-231, SK-BR-3 and CRL-1500) and one normal breast epithelium cell line (HMEC). Although HMEC is normal mammary gland cell line, it was immortalized and expressed GGT protein that was not expressed in normal mammary gland *in vivo*. Indeed, all five cell lines expressed GGT protein and large fluorescence increase was detected by means of fluorescence microscopy in all five-cell lines after application of gGlu-HMRG in the culture medium (Fig. 1a,b for MCF7; Supplementary Fig. 1 for the others). In accordance with our previous findings^10^, fluorescence was detected within 20 minutes after gGlu-HMRG administration and the signal continued to increase time-dependently (Fig. 1c). Further, the use of siRNA to knock down GGT blocked the fluorescence increase (Fig. 1b,c).

Next, we topically applied gGlu-HMRG to freshly excised human breast specimens containing various lesions together with normal tissues. We found that tumorous lesions exhibited a time-dependent increase of green fluorescence that clearly distinguished them from surrounding mammary gland and fat (Fig. 1d–f). Although normal breast tissues show auto-fluorescence, its color was different from that generated by gGlu-HMRG, and its intensity remained constant during incubation. Therefore, tumorous lesions could be clearly identified by observing the change of fluorescence intensity. Comparison of the fluorescence images with cell-by-cell histopathological mapping in hematoxylin and eosin (HE)-stained images confirmed that highly fluorescent regions coincided with tumorous lesions (Fig. 1g,h). Even tiny tumors only a few hundred micrometers in size, such as ductal carcinoma *in situ* (DCIS), were detected by gGlu-HMRG (Fig. 1h). Based on these results, we next measured the fluorescence increase (FI) during 5 min incubation of gGlu-HMRG with various breast lesions and adjacent normal tissues, using specimens excised from 35 patients. The clinicopathological data and FI for 36 lesions (one patient showed two lesions) are shown in Supplementary Table 1. Abnormal (malignant and proliferative) lesions had significantly higher FI than normal tissues (Supplementary Table 2). When we set a threshold value of 8.0 a.u., the sensitivity and specificity for binary classification (normal/abnormal) were 92% and 94%, respectively (Fig. 1i, and area under curve is shown in Supplementary Fig. 2). Though BC is known to consist of a number of distinct pathological entities and to exhibit molecular heterogeneity^11^, gGlu-HMRG was able to detect a broad range of BC.

We also examined the samples immunohistochemically to confirm protein expression of GGT by breast cancer cells. We found that GGT was expressed in the membrane (Supplementary Fig. 3) in the majority of breast malignant lesions and proliferative lesions examined (positive rates were 84% and 83%, respectively). Normal breast tissues showed uniformly negative staining for GGT (0%, Supplementary Table 3).

Since these results indicated that the gGlu-HMRG fluorescent method might indeed be suitable for intraoperative pathological margin assessment, we next applied it to examine malignant lesions in the margin of BCS specimens (N = 7 from 5 patients). We compared the fluorescence-positive areas with the malignant lesions independently identified by pathologists. All of the identified malignant lesions showed up as fluorescence-positive areas (Fig. 2 and Supplementary Fig. 4). These results support the validity of this fluorescence-based method as a new clinical tool for pathologists to identify candidate tumorous and proliferative lesions for pathological confirmation. From another point of view, gGlu-HMRG could be useful to identify regions containing no malignancy as fluorescence increase-negative regions (Fig. 2 and Supplementary Fig. 4 and 5), where intraoperative microscopic examination by pathologists would not be required.

A limitation of this method is the bright fluorescence of benign regions and proliferative lesions such as mastopathy and hyperplasia; in other words, the method cannot distinguish malignant and benign regions. Therefore, it would be necessary for pathologists to diagnose all fluorescence-positive regions during BCS.

Despite this limitation, this fluorescent method has high sensitivity and space resolution. It could help not to miss minute malignant lesions such as several hundred μm sized extensive intraductal component exposed on cross-section specimen which we could not find macroscopically (Fig. 2g–k), and contribute to complete tumor resection in cancer surgery. We believe the gGlu-HMRG method represents a breakthrough in intraoperative margin assessment, making it possible to evaluate total-circumferential cross-section surfaces that are too large for pathologists to assess microscopically. Further, evaluation can be done within only 5 minutes after probe application. Since it is simple and non-tissue-destructive, it could be readily adopted in the clinical setting. We anticipate that it will reduce costs and the burden on histopathology services, and contribute to decreasing the margin-positive rate in BCS, thereby improving patient outcome.

## Materials and Method

### Clinical Samples

**Figure 1:**
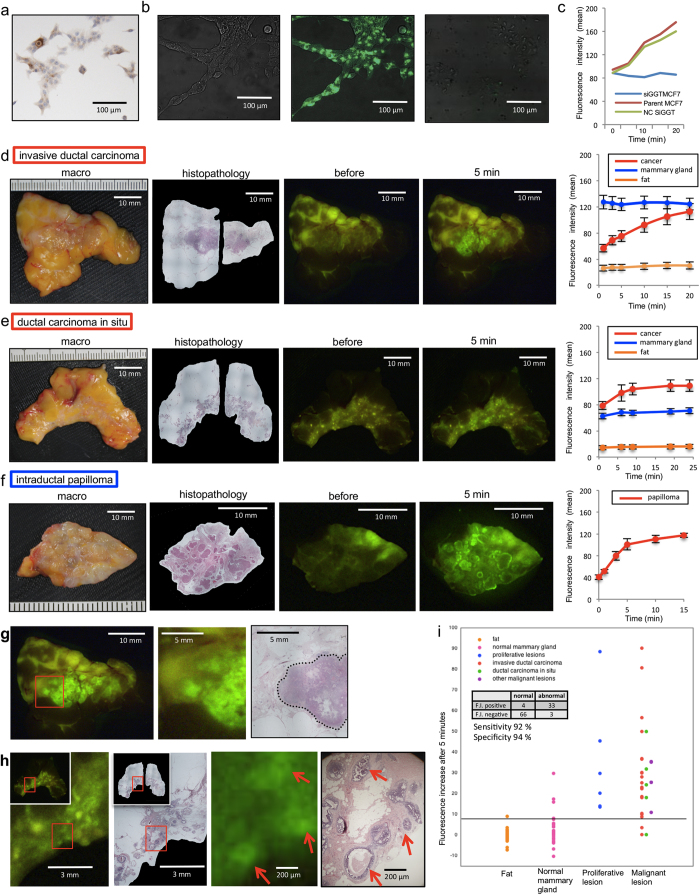
(**a**) Immunohistochemical staining of MCF-7 cell line for GGT. (**b**) Fluorescence image of MCF-7 cells obtained after administration of gGlu-HMRG. A differential interference contrast (DIC) image (left), a gGlu-HMRG fluorescence image (middle), and an image of cells pretreated with siRNA targeting GGT before application of gGlu-HMRG (right). (**c**) Time-dependent change of fluorescence intensity in MCF-7 cell line and MCF-7 cell line pretreated with siRNA targeting GGT. (**d**~**f**) Time-dependent fluorescence images of various breast tumor specimens [invasive ductal carcinoma (**d**), DCIS (**e**), intraductal papilloma (**f**)] after administration of gGlu-HMRG probe. In each of the specimens shown in (**d**–**f**), the time-dependent fluorescence intensities were measured at tumor lesions, normal mammary gland regions and fat (right column). (**g**, **h**) Comparisons of fluorescence localization (**d** and **e**) with pathological HE staining of the same specimen. (**g**) The cancer region is enclosed by a dotted line in the HE-staining image. Areas of increased fluorescence coincided well with pathologically cancerous region. Red arrows in (**h**) show fluorescence-positive area and malignant lesions. (**i**) Comparison of fluorescence increases (FI) after administration of gGlu-HMRG in breast lesions and normal tissues.

**Figure 2:**
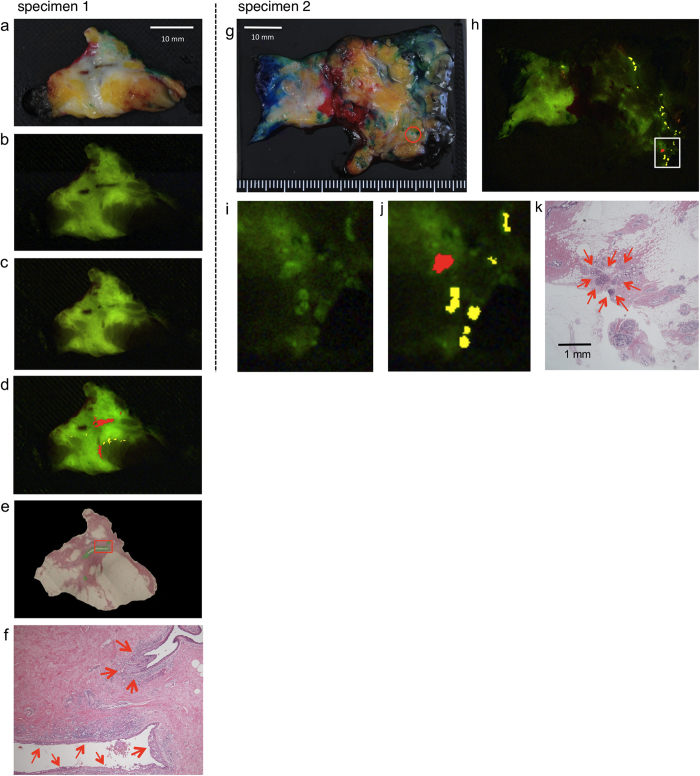
Application of the gGlu-HMRG fluorescence method in surgical margins of BCS specimens. Specimen 1 was diagnosed pathologically as DCIS and specimen 2 was diagnosed as invasive ductal carcinoma (papillotubular). (**a**) Gross picture. (**b**) Fluorescence image just before gGlu-HMRG administration. (**c**) Fluorescence image 5 minutes after gGlu-HMRG administration. Increased brightness was observed in some areas. (**d**) Red and yellow colors indicate fluorescence-positive areas. Red areas were identified as malignant lesions. Yellow areas were fluorescent positive and did not identified as malignant lesions. (**e**) HE stained image after formalin fixation. Malignant regions identified from pathological findings are colored green. (**f**) The area in the red box of (**e**) is magnified. Red arrows showed malignant lesions in the cross section of surgical margin. (**g**) Gross picture. Red-circled areas showed malignant lesions diagnosed from pathological findings. (**h**–**j**) Red and yellow areas showed fluorescence-positive areas. Red areas were fluorescent positive and identified as malignant lesions. Yellow areas were fluorescent positive and did not identified as malignant lesions. The area in the white box of (**h**) is magnified in (**i**) and (**j**). **i**) The picture at 5 minutes after gGlu-HMRG adminstration. (**j**) After analyzing the picture, we found and colored fluorescent positive area as red and yellow. (**k**) HE-staining image same region as (**i**) and (**j**) after formalin fixation. Red arrows indicated extensive intraductal components of invasive ductal carcinoma.

Seventy-two breast cancer patients underwent surgical treatment at the Kyushu University Beppu Hospital and the Ueo Breast Surgery Clinic during 2012 to 2013. Thirty-five patients were enrolled for discriminative analysis for various tissues in breast (Fig. 1i) and 49 patients were enrolled for immunohistochemical analysis (Supplementary Table 3) and 5 patients were enrolled for margin assessment (Fig. 2 and Supplementary Fig. 4 and 5). Some patients were duplicated in plural analyses. Informed consent was obtained from all patients, and this study was approved by the ethics committees of Kyushu University and the local ethics committees. All experiments were performed in accordance with guidelines and regulations approved by ethics committees. Tumor specimens were taken intraoperatively and fluorescence images were collected with an in-house made portable fluorescence camera in NIH for detecting this GGT-activatable probe^12^ every five minutes after gGlu-HMRG administration. Specimens were fixed in formalin for pathologic examination. BC was diagnosed according to the clinicopathological criteria of the Japan Breast Cancer Society. All clinical and pathologic data were obtained from medical records.

### Cell lines and culture

Five established human breast cancer cell lines were used in this study: MDA-MB-231, MCF7, CRL-1500, SK-BR-3 (provided by the Cell Resource Center of Biomedical Research, Institute of Development, Aging and Cancer, Tohoku University) and HMEC (provided by LifeLine). MCF7, CRL-1500, SK-BR-3 were grown in RPMI 1640 medium (Life Technologies) containing 10% fetal bovine serum (Life Technologies), 0.03% L-glutamine at 37 °C, penicillin (100 U/ml), and streptomycin (100 μg/ml) in 5% CO_2_. MDA-MB-23 was grown in Dulbecco’s modified Eagle’s medium (DMEM, Life Technologies) containing 10% fetal bovine serum (Life Technologies), 0.03% L-glutamine at 37 °C, penicillin (100 U/ml), and streptomycin (100 μg/ml) in 5% CO_2_. HMEC was grown in MammaryLife^TM^ Basal Medium (LifeLine) containing MammaryLife LifeFactors kit (LS-1088, LifeLine), penicillin (100 U/ml), and streptomycin (100 μg/ml) in 5% CO_2_.

### *In vitro* fluorescence microscopy

The change of fluorescence intensities of MDA-MB-231, MCF7, CRL-1500, and SK-BR-3 cells after gGlu-HMRG administration *in vitro* was examined by fluorescence microscopy. gGlu-HMRG (10 μM, containing 0.5% v/v DMSO as a co-solvent) in RPMI1640 was added to the culture medium and the cells were further incubated. 1 × 10^6^ cells from each cell line were plated on a cover glass-bottomed culture well and incubated with 1 × 10^−5^M gGlu-HMRG at 37 °C. Fluorescent images were captured every 5 minutes within 30 minutes with a confocal LSM510 microscope and Carl Zeiss Micro imaging utilized excitation from a 488 nm argon ion laser line and emission after passing through 505–530 band-pass filters (LP505). The laser power was 5% of 25 mW, detector gain was 534 V and acquisition time was 4 seconds. Transmitted light differential interference contrast (DIC) images were also acquired. Knock-down of GGT in cell lines with siRNA was carried out as previously described^10^.

### Fluorescence imaging study in clinical specimens

We drop 3 ml gGlu-HMRG (50 μM, containing 0.5% v/v DMSO as a co-solvent) in RPMI1640 onto the specimens on the slides. To evaluate the generation of fluorescence from gGlu-HMRG in various tissues, slices of specimens were placed on black dishes in an in-house fluorescence camera unit. Snapshot images were used for calculating fluorescence intensities. Each picture was recorded in pixel intensity values in the range from 0 to 255.

To evaluate the fluorescence of various tissues, circular regions of interest (ROIs) were set in the center of representative lesions and normal tissues chosen according to the pathological diagnosis based on examination of hematoxylin and eosin-stained specimens after formalin fixation. The number of ROIs drawn in normal regions was not equal to that in abnormal regions, because tumorectomy did not afford substantial amounts of normal breast tissues. We calculated the average fluorescence intensity of each ROI and determined the fluorescence increase by subtracting the mean intensity just after gGlu-HMRG administration from that at 5 minutes post application. All fluorescence images were analyzed with Image J software (National Institutes of Health, Rockville, Maryland, USA) (http://rsbweb.nih.gov/ij/).

For evaluation of FI in surgical margins of BCS, fluorescence-positive areas were identified in a semi-automatic manner using the commands of ‘Image J’. Areas having a specified FI and amplitude were extracted by subtraction and use of the particle analysis function. Areas with an FI of more than 8.0 a.u. and at least 20 pixels in size were designated as fluorescence-positive areas.

### Histological analysis

Excised specimens were immediately fixed with 10% formaldehyde for at least 24 hours. Paraffin-embedded sections were stained with hematoxylin and eosin for histopathological evaluation. Experienced pathologists examined each sample in a blinded manner, and dysplasia and neoplasia were diagnosed according to the Japanese Breast Cancer Society criteria and WHO Classification of Tumors of the Breast, 4th edition.

### Immunohistochemical analysis

The clinicopathological findings for breast tissue samples used for immunohistochemical examination are shown in Supplementary Table 3. Slides were deparaffinized in xylene, sequentially washed in 100%, 90% and 80% of ethanol, and then washed in TBS. After heat-induced antigen retrieval (citrate, pH 6), each slide was pre-incubated in 1% H_2_O_2_ followed by incubation in blocking solution (normal horse serum) for 2 hours. Then the slide was incubated overnight with primary antibody GGT1 (sc-100746, Santa Cruz Biotechnology, Inc., California, USA) at 4 °C at a dilution of 1:200, processed according to the polymer method (EnVision™ + Kits; Dako, Kyoto, Japan), and counterstained with hematoxylin. 3,3′-Diaminobenzidine tetrahydrochloride (DAB) reaction time was 30 seconds. When no stained cell was found, we evaluated the tissues as negative, and otherwise we evaluated them as positive.

### Statistical analysis

Statistical analyses were carried out using JMP software (SAS Institute Inc., Cary, NC). The Mann-Whitney test was used to compare mean fluorescence intensity values. P value ≤ 0.05 was considered to indicate a statistically significant difference.

## Additional Information

**How to cite this article**: Ueo, H. *et al.* Rapid intraoperative visualization of breast lesions with γ-glutamyl hydroxymethyl rhodamine green. *Sci. Rep.* **5**, 12080; doi: 10.1038/srep12080 (2015).

## Supplementary Material

Supplementary Information
