# Supplementary material for: Rapid intraoperative visualization of breast lesions with γ-glutamyl hydroxymethyl rhodamine green
Source: Sci Rep. 2015 Jul 13;5:12080. doi: 10.1038/srep12080 (PMC4499838; doi:10.1038/srep12080)
Supplement: Supplementary Information [file srep12080-s1.pdf]

Rapid intraoperative visualization of breast lesions with γ-glutamyl hydroxymethyl rhodamine green

Hiroki Ueo, Yoshiaki Shinden, Taro Tobo, Ayako Gamachi, Mitsuaki Udo, Hisateru Komatsu, Sho Nambara, Tomoko Saito, Masami Ueda, Hidenari Hirata, Shotaro Sakimura, Yuki Takano, Ryutaro Uchi, Junji Kurashige, Sayuri Akiyoshi, Tomohiro Iguchi, Hidetoshi Eguchi, Keishi Sugimachi, Yoko Kubota, Yuichiro Kai, Kenji Shibuta, Yuko Kijima, Heiji Yoshinaka, Shoji Natsugoe, Masaki Mori, Yoshihiko Maehara, Masayo Sakabe, Mako Kamiya, John W. Kakareka, Thomas J. Pohida, Peter L. Choyke, Hisataka Kobayashi, Hiroaki Ueo, Yasuteru Urano and Koshi Mimori

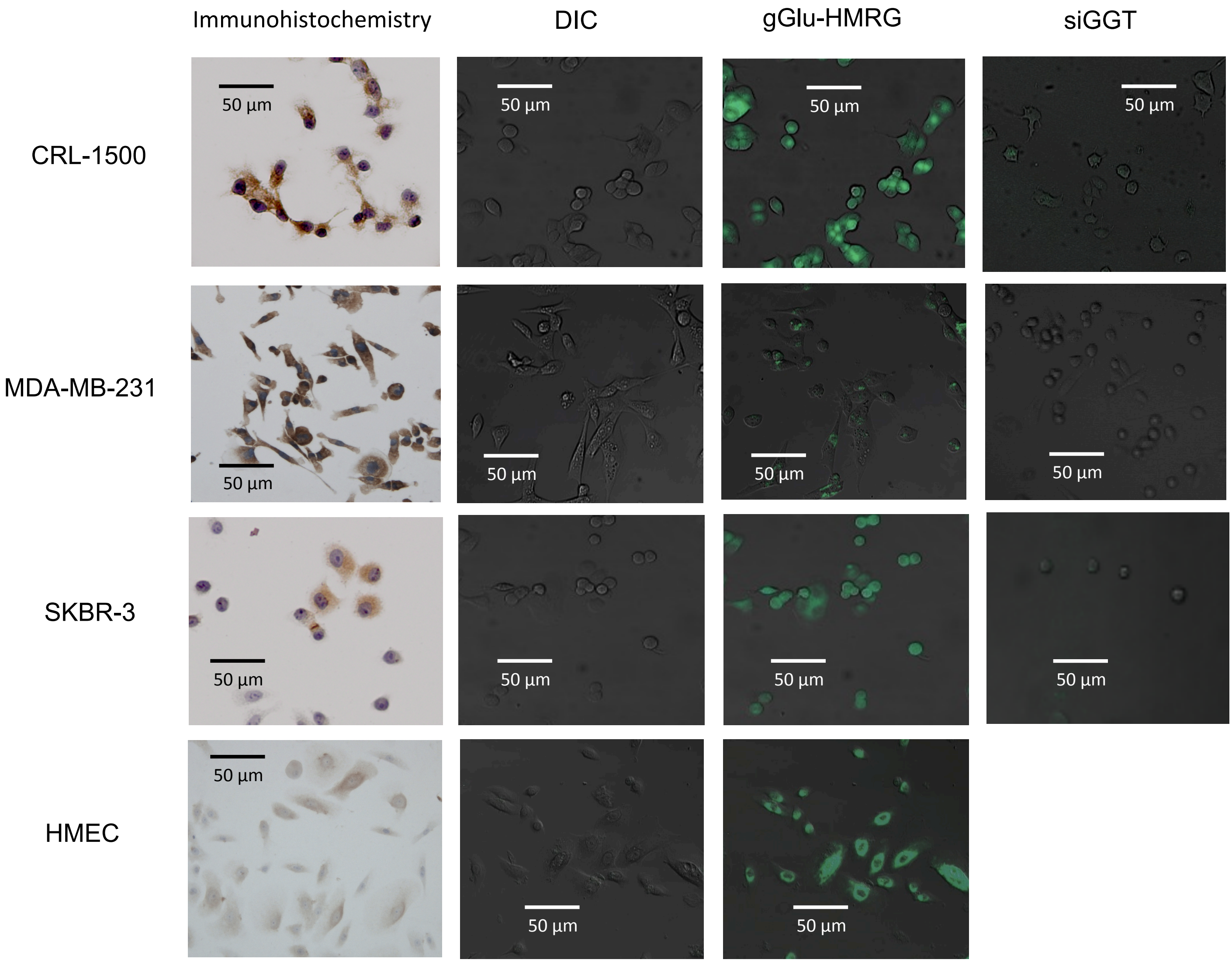

Supplementary Figure 1: Immunohistochemical images for GGT (left) and fluorescence images of breast cancer cell lines and normal breast epithelial cell obtained in the presence of gGlu-HMRG. Differential interference contrast (DIC) images (second from the left), gGlu-HMRG fluorescence images (second from the right), and images of cells pretreated with siGGT before gGlu-HMRG (right).

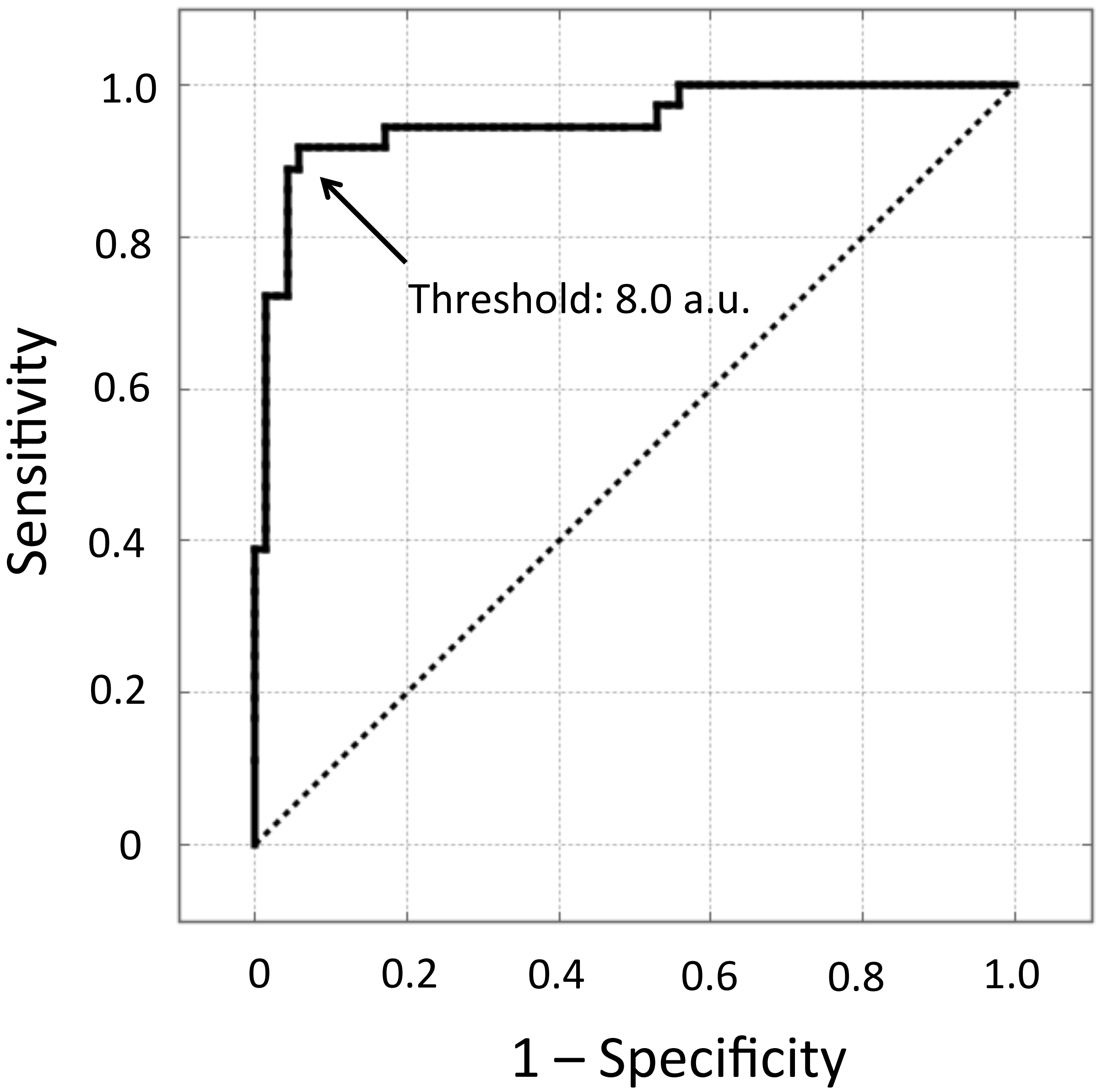

Supplementary Figure 2: Area under the curve for fluorescent method is shown. The point of threshold we set is shown by arrow.

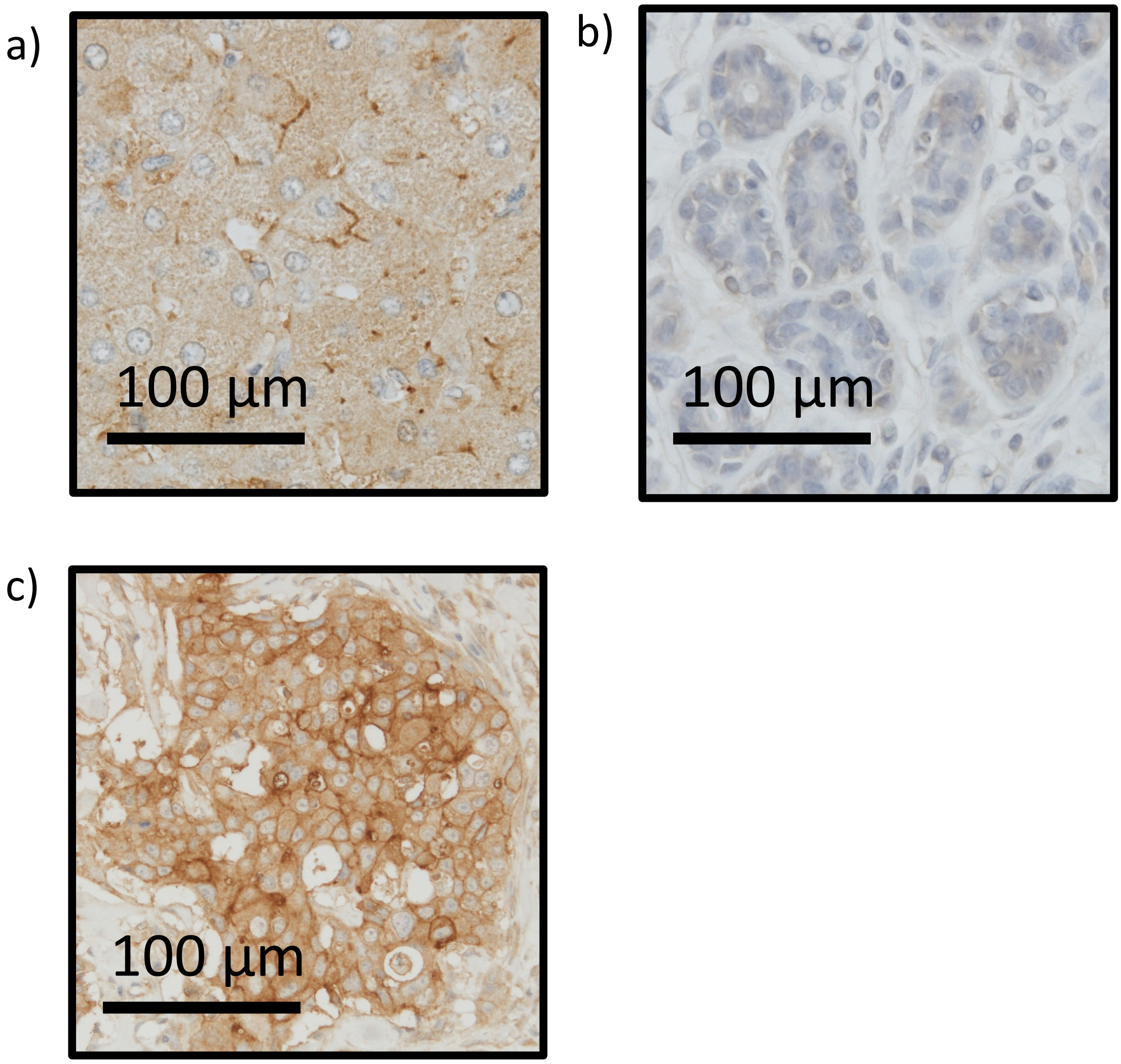

Supplementary Figure 3: Immunohistochemical staining of human breast cancer tissue for GGT. GGT is expressed in the membrane of cells. a) Positive control for liver. b) Negative control for normal duct epithelial cells in the breast. c) An example of breast cancer cells positive for GGT antibody.

Clinicopathological information of cases validated by fluorescence method

			n
Age		61 (29-86)	
Estrogen receptor		positive	22
		negative	7
		unnknown*1	1
Progesterone receptor		positive	18
		negative	11
		unknown*1	1
HER2 expression		positive	3
		negative	21
		unknown*2	6
Pathological features			
Malignant lesions			30
	Invasive ductal carcinoma		20
		scirrhous	7
		solid-tubular	4
		papillo-tubular	7
		mixed and unknown	2
	DCIS		5
	Invasive lobular carcinoma		1
	Apocrine carcinama		1
	Medullary carcinoma		1
	Recurrent lesion		2
Proliferative lesions			6
	Papilloma		3
	Sclerosing adenosis		1
	Hyperplasia		1
	Mastopathy		1
			35*3

*1 Estrogen receptor status was unknown in one case of skin recurrence.
*2 HER2 status was unknown in one case of skin recurrence and 5 cases of ductal carcinoma in situ (DCIS).
*3 One case contained both malignant lesion and proliferative lesion.

Fluorescence-positive rate and mean value of fluorescence increase in various breast tissues

	n	FP*1 rate	mean value of FI*2	
**Malignant lesions**	**30**	**90%**	**28.6**	
**Invasive ductal carcinoma**	**20**	**85%**	**30**	
**DCIS**	**5**	**80%**	**22.8**	
**Invasive lobular carcinoma**	**1**	**100%**	**25.3**	
**Other carcinoma**	**4**	**100%**	**26.7**	
**Proliferative lesions**	**6**	**100**	**33.8**	$p=0.60$
**Normal mammary gland**	**35**	**9%**	**2.1**	$p<0.0001$
**Fat**	**35**	**3%**	**-0.2**	$p<0.0001$

*1 FP: fluorescence-positive
*2 FI: fluorescence increase

Immunohisotochemical analysis of GGT expression in human breast tissues

Histopathological classification	positive rate	
**Malignant lesions**	**84%**	**(36/43)**
Invasive ductal carcinoma	91%	(29/32)
papillo-tubular	85%	(11/13)
solid-tubular	88%	(7/8)
schirrhous	100%	(10/10)
mixed	100%	(1/1)
Lobular carcinoma	100%	(1/1)
Other carcinoma	75%	(3/4)
DCIS*	50%	(2/4)
Metastatic lesion	50%	(1/2)
**Proliferative lesions**	**83%**	**(5/6)**
Papilloma	67%	(2/3)
Adenosis	100%	(1/1)
Fibroadenoma	100%	(1/1)
Mastopathy	100%	(1/1)
**Normal tissues**	**0%**	**(0/40)**
Mammary gland	0%	(0/20)
Fat	0%	(0/20)

*DCIS: ductal carcinoma in situ

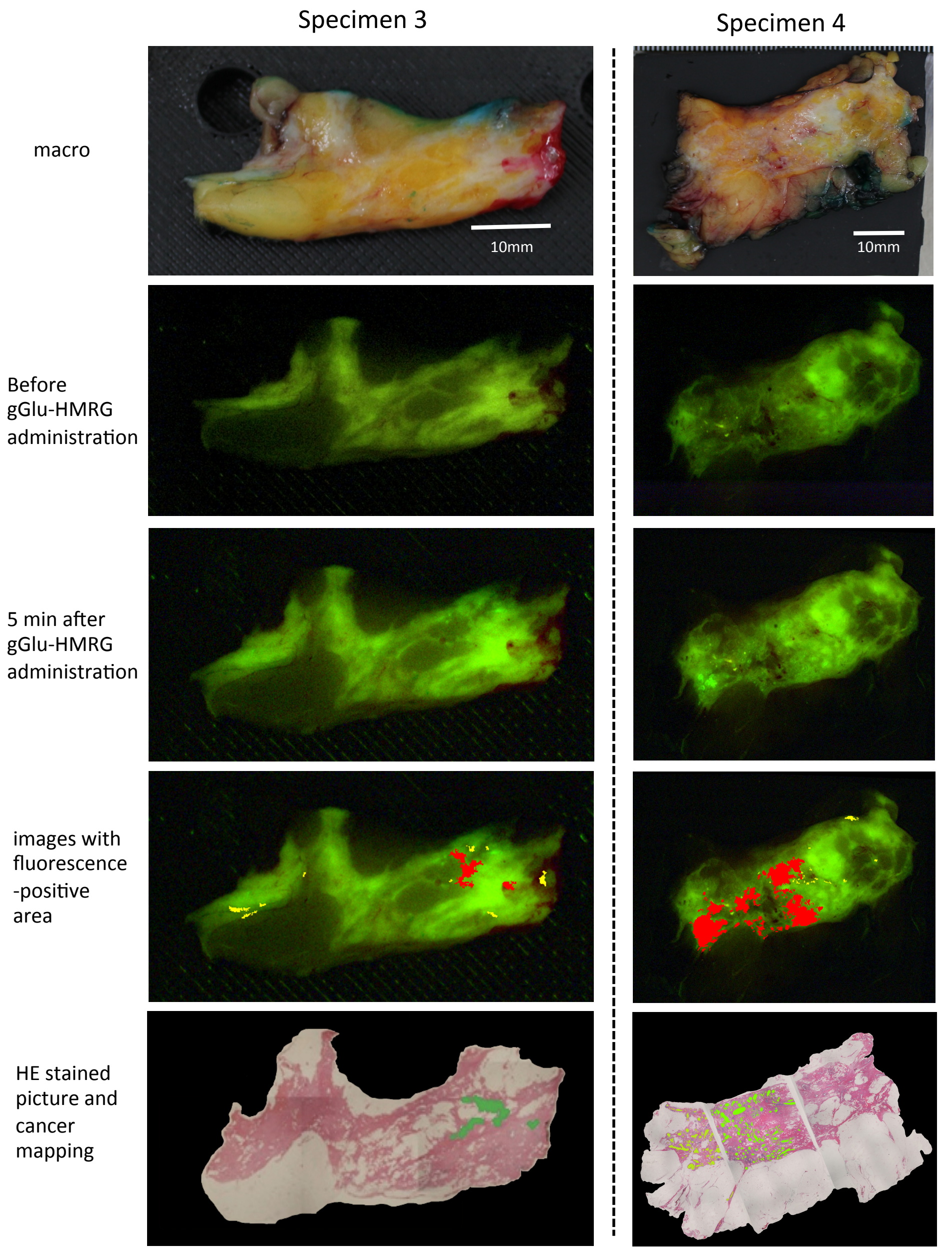

Supplementary Figure 4: Application of gGlu-HMRG fluorescence method to evaluate BCS specimens in which the surgical margins were malignancy - positive. Specimen 3 was diagnosed pathologically as DCIS and specimen 4 was also diagnosed as DCIS. 1st line; gross pictures. 2nd line; fluorescence images just before gGlu-HMRG administration. Auto-fluorescence of mammary gland appears as green. 3rd line; fluorescence images 5 minutes after application of gGlu-HMRG. Increased fluorescence was observed in some areas. Using the images in the 2nd and 3rd lines, the fluorescence-positive areas were identified with Image J software. In the 4th line, calculated fluorescence-positive areas were shown in red and yellow on the images from the 3rd line. Red indicates malignant regions in fluorescence-positive areas. 5th line; HE-staining images after formalin fixation. Based on the pathological findings, malignant regions are colored green. All pathologically identified malignant lesions were fluorescence - positive.

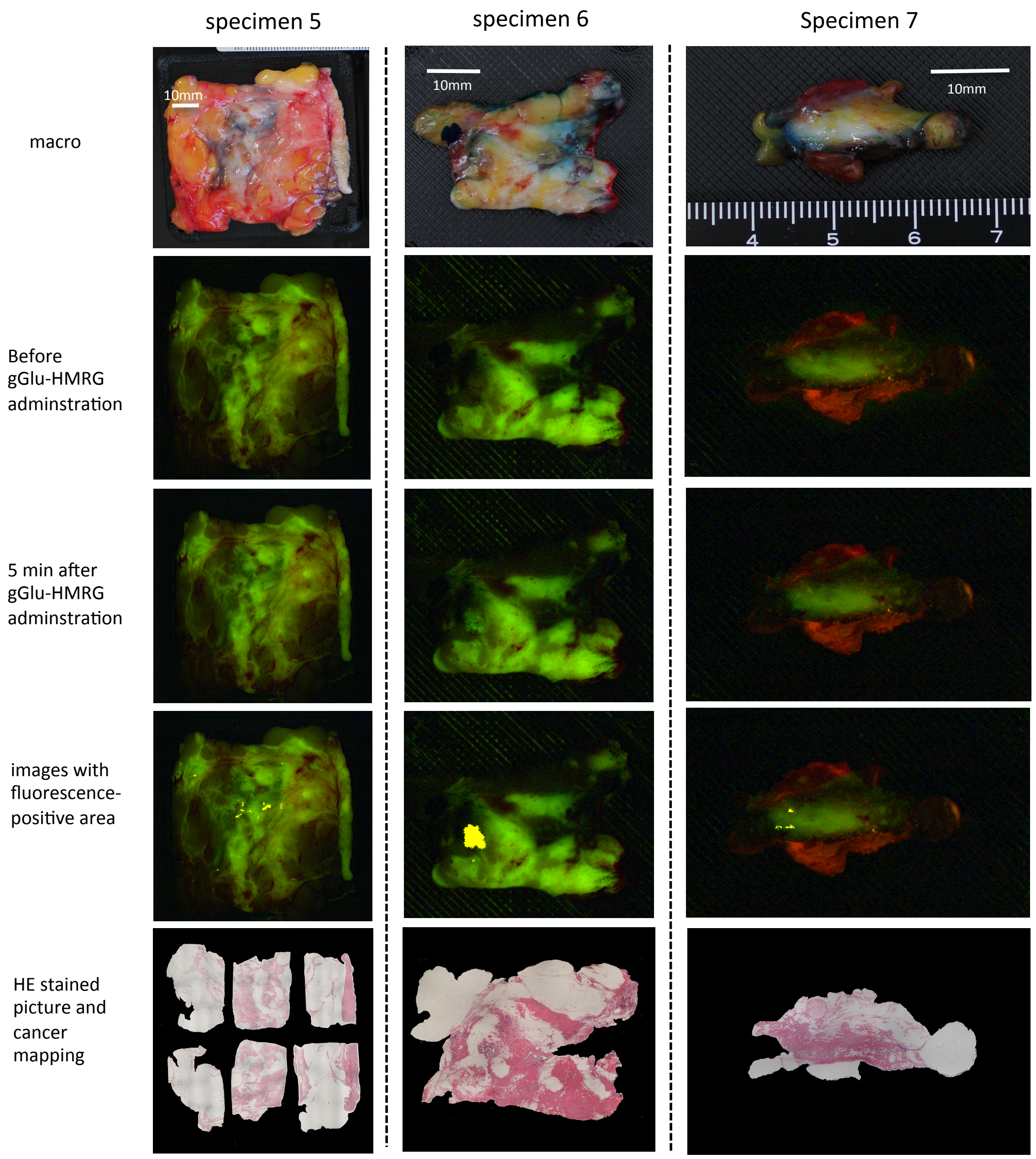

Supplementary Figure 5: Application of the gGlu-HMRG fluorescent to BCS specimens in which the surgical margins were malignancy-negative. Specimen 5 was diagnosed pathologically as invasive ductal carcinoma (schirrhous), specimen 6 as DCIS, and specimen 7 as invasive ductal carcinoma (papillotubular). Images in each column correspond to those in Supplementary Figure 3. In the 4th line, calculated fluorescence-positive areas at 5 minutes after gGlu-HMRG administration are indicated as yellow areas. No malignant regions were detected in these specimens (bottom lines).
